# Spatial Patterns in Rush-Hour vs. Work-Week Diesel-Related Pollution across a Downtown Core

**DOI:** 10.3390/ijerph15091968

**Published:** 2018-09-10

**Authors:** Brett J. Tunno, Drew R. Michanowicz, Jessie L. C. Shmool, Sheila Tripathy, Ellen Kinnee, Leah Cambal, Lauren Chubb, Courtney Roper, Jane E. Clougherty

**Affiliations:** 1 Department of Environmental and Occupational Health, Graduate School of Public Health, University of Pittsburgh, Pittsburgh, PA 15261, USA; drewmichanowicz@gmail.com (D.R.M.); jlcshmool@gmail.com (J.L.C.S.); sheila.tripathy@gmail.com (S.T.); ejk40@pitt.edu (E.K.); ltipton86@gmail.com (L.C.); lgc4@pitt.edu (L.C.); clr56@pitt.edu (C.R.); jec373@drexel.edu (J.E.C.); 2 Dornsife School of Public Health, Drexel University, Philadelphia, PA 19104, USA

**Keywords:** air pollution monitoring, black carbon (BC), fine particulate matter (PM_2.5_), geographic information systems (GIS), land use regression (LUR), organic compounds, trace elements

## Abstract

Despite advances in monitoring and modelling of intra-urban variation in multiple pollutants, few studies have attempted to separate spatial patterns by time of day, or incorporated organic tracers into spatial monitoring studies. Due to varying emissions sources from diesel and gasoline vehicular traffic, as well as within-day temporal variation in source mix and intensity (e.g., rush-hours vs. full-day measures), accurately assessing diesel-related air pollution within an urban core can be challenging. We allocated 24 sampling sites across downtown Pittsburgh, Pennsylvania (2.8 km^2^) to capture fine-scale variation in diesel-related pollutants, and to compare these patterns by sampling interval (i.e., “rush-hours” vs. “work-week” concentrations), and by season. Using geographic information system (GIS)-based methods, we allocated sampling sites to capture spatial variation in key traffic-related pollution sources (i.e., truck, bus, overall traffic densities). Programmable monitors were used to collect integrated work-week and rush-hour samples of fine particulate matter (PM_2.5_), black carbon (BC), trace elements, and diesel-related organics (polycyclic aromatic hydrocarbons (PAHs), hopanes, steranes), in summer and winter 2014. Land use regression (LUR) models were created for PM_2.5_, BC, total elemental carbon (EC), total organic carbon (OC), elemental (Al, Ca, Fe), and organic constituents (total PAHs, total hopanes), and compared by sampling interval and season. We hypothesized higher pollution concentrations and greater spatial contrast in rush-hour, compared to full work-week samples, with variation by season and pollutant. Rush-hour sampling produced slightly higher total PM_2.5_ and BC concentrations in both seasons, compared to work-week sampling, but no evident difference in spatial patterns. We also found substantial spatial variability in most trace elements and organic compounds, with comparable spatial patterns using both sampling paradigms. Overall, we found higher concentrations of traffic-related trace elements and organic compounds in rush-hour samples, and higher concentrations of coal-related elements (e.g., As, Se) in work-week samples. Mean bus density was the strongest LUR predictor in most models, in both seasons, under each sampling paradigm. Within each season and constituent, the bus-related terms explained similar proportions of variance in the rush-hour and work-week samples. Rush-hour and work-week LUR models explained similar proportions of spatial variation in pollutants, suggesting that the majority of emissions may be produced during rush-hour traffic across downtown. Results suggest that rush-hour emissions may predominantly shape overall spatial variance in diesel-related pollutants.

## 1. Introduction

Despite improvements in monitoring and modelling of intra-urban variation in multiple air pollutants, few studies have attempted to separate spatial patterns by time of day, or to incorporate organic tracers for key emissions sources [1]. Because diesel particulate matter (DPM) is a likely carcinogen [2] and is linked to other health outcomes [3,4,5], more clearly identifying diesel emissions sources in densely-populated areas can help to identify opportunities to reduce exposures, which may lead to substantial improvements in population health.

Many studies have captured fine-scale spatial variation in urban air pollution, and successfully applied land use regression (LUR) and related spatial modelling methods to estimate exposures and to support identification of key sources [6,7,8]. Due to logistical and technical limitations related to deploying a large number of non-programmable monitors simultaneously, however, few studies have been able to perform spatial sampling specific to key hours of interest (e.g., rush hours, inversion hours) [9].

Further, although there have been substantial improvements in methods for multi-pollutant saturation monitoring [10], relatively few spatial studies have incorporated source-specific organic particle components [11,12,13], in part due to the greater volatility and instability of these compounds. To capture diesel-related sources, however—and particularly to attempt to separate multiple diesel sources (e.g., trucks vs. buses)—multiple organic markers are normally necessary [6,8,9,14,15].

Downtown Pittsburgh (PA, USA) is a compact urban core (~2.8 km^2^) impacted by a large number of diesel-related sources (i.e., buses, trucks, diesel-engine barges, construction activity). We monitored 24 spatially-distributed sites, summer and winter, for a wide suite of pollutants (i.e., fine particles (PM_2.5_), black carbon (BC), elemental carbon (EC), organic carbon (OC), a suite of polycyclic aromatic hydrocarbons (PAHs), hopanes, steranes, and trace elements). At each site, paired samplers collected samples for the full work week (Monday–Friday, 7 a.m.–7 p.m.), or solely during high-diesel hours (Monday–Friday, 5 a.m.–10 a.m. and 3 p.m.–7 p.m.). We compared spatial variation by sampling interval and season, and developed season- and interval-specific LUR models. We hypothesized higher pollution concentrations and greater spatial contrast during rush-hour sampling, with variation by season and pollutant. This study is the first, to our knowledge, to capture and compare spatial variation in multiple pollutants, including organic components, during high-diesel vs. other work-week hours.

## 2. Materials and Methods

We systematically allocated twenty-four (24) air monitoring sites, using previously-developed geographic information system (GIS)-based methods, to characterize variation in total traffic, buses, and truck traffic across our small domain [1]. The same sites were sampled in both summer and winter, and were randomly distributed over four 5-day (Monday through Friday) sampling sessions. At each site, we collected paired work-week and rush-hour samples for a suite of gasoline- and diesel-related pollutants. Temporally-adjusted concentrations at each site, for each season, were derived using time trends observed at two reference monitors (one urban and one rural background site). This study design is further detailed, and maps of the reference sites provided, in Tunno et al., [1].

### 2.1. Study Domain

**Figure 1:**
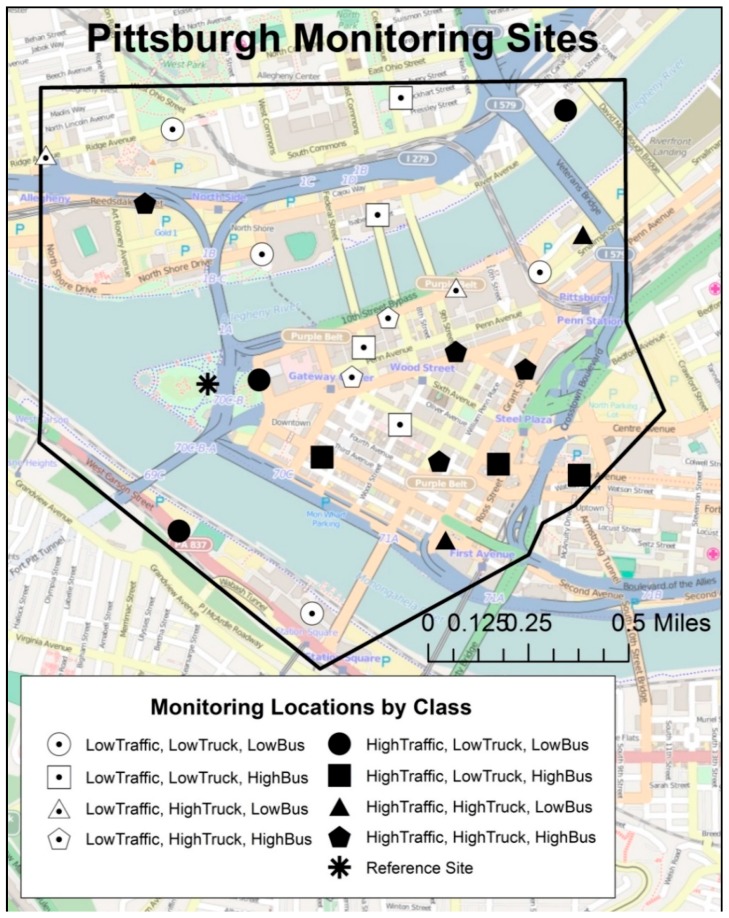
Downtown Pittsburgh monitoring locations (*n* = 24) and reference site by class dichotomization (total traffic density, truck traffic density, bus route density). Rural upwind reference site to the west (~14.5 km) of the study area is not shown.

We used GIS to fit a polygon that included downtown Pittsburgh, with a uniform elevation of roughly 300 m, to encompass vehicular traffic across highways and bridges surrounding the downtown core, resulting in a sampling domain of roughly 2.8 km^2^ (Figure 1).

### 2.2. Tracer Selection

We identified chemical tracers for key pollution sources of interest (i.e., heavy trucks, buses, and gasoline vehicles). We collected integrated samples of PM_2.5_, BC, trace elements, and organic compounds (PAHs, hopanes, steranes). We also identified elemental constituents found to be associated with diesel emissions in at least two published studies (Al [16,17], Ca [18,19,20], and Fe [20,21]), based on our previous literature search, and included additional elements related to vehicular emissions, brake/tire wear, soil/road dust resuspension, steel-making, and coal [22]. We selected organic markers previously identified in diesel exhaust, quantifiable using thermal desorption gas-chromatography mass-spectrometry (TD-GC-MS) [15], and relatively lower volatility and reactivity relative to other components of diesel exhaust [9]. Additional information on organic compound marker selection and quartz filter handling is detailed in Tunno et al. [1].

### 2.3. Sampling Intervals

Paired samplers ran two (2) programs: one to capture full work-week concentrations (Monday through Friday, 7 a.m. to 7 p.m.), and one to capture rush-hours (Monday through Friday, 5 a.m. to 10 a.m. and 3 p.m. to 7 p.m.). These “rush-hours” were identified as the hours with the heaviest bus and truck traffic, based on the Pennsylvania Department of Transportation (PennDOT) hourly traffic counts and Allegheny County Port Authority bus schedules for the downtown area. Winter sampling was performed from 13 January to 7 February, and summer sampling from 7 July to 1 August 2014.

### 2.4. Instrumentation

We used programmable, portable ambient air sampling units [23] which included Harvard Impactors (HI) (Air Diagnostics and Engineering, Inc., Harrison, ME, USA) with 37 mm Teflon filters (PTFE membrane, 2 µm pores, Pall Life Sciences (Port Washington, NY, USA), and HOBO data loggers for temperature and relative humidity (Onset Computer Corporation, Bourne, MA, USA). Battery-operated vacuum pumps (SKC, Inc., Eighty-Four, PA, USA) maintained a flow rate of 4 L per minute (LPM). Particle sampling instruments were housed in weather-tight Pelican boxes.

We adapted these sampling units to collect integrated samples of organic compounds. In separate weather-tight Pelican boxes, additional PM_2.5_ samples to analyze for organic compounds were collected using cyclones (Air Diagnostics and Engineering, Inc., (Harrison, ME, USA)) using 37 mm quartz fiber filters (Pallflex Tissuquartz non-heat treated filters, Pall Life Sciences), pre-baked at 900 deg F prior to sampling to remove VOC contamination which may interfere with collection of organic particle components. All samplers were mounted approximately 3 m above ground, near the human breathing zone, but sufficiently out-of-reach to avoid tampering. Most importantly, the sampling height was consistent across all sites, for comparability. Organics samplers were strictly deployed on metal poles, to avoid contamination by VOCs from treated wooden poles [24].

To enable comparisons of elemental vs. organic, and rush-hour vs. work-week, samples, four (4) monitors were deployed together. Two sampling units collected PM_2.5_, BC, and trace elements on Teflon filters using Harvard Impactors (HIs); two additional units collected organic compounds using cyclones. One unit of each type was programmed to capture work-week hours, and the other to capture only rush hours. Because four sampling units could not be placed on the same utility pole without obstructing each other, we selected, as needed, the closest suitable pole for the HI (Teflon) samplers, and used the original pole for the cyclone (quartz) samplers.

### 2.5. Site Selection and Allocation

We aimed to capture the spatial variability in traffic, particularly with regard to diesel- and gasoline-powered vehicles, by allocating sites using three GIS-based indicators: total traffic density, total truck density, and bus route frequency, as described in Tunno et al. [1]. To compare rush-hour and work-week paired samples, we modified the original site allocation to enable paired sampling. For phase 2, we retained 24 distributed sites from the original 36 sites (66%) for spatial and source variation (Figure 1). Sites were eliminated (*n* = 12 sites) based on similarity in temporally-adjusted organic concentrations with those detected at nearby sampling locations during the Phase 1 summer and winter campaigns [1], to maximize the retained spatial variation in concentrations across the study area. We retained two reference sites (one upwind background site, one urban site within the domain).

### 2.6. Laboratory Analyses:

Using an ultramicrobalance (Mettler Toledo Model XP2U, Columbus, OH, USA), Teflon filters were pre- and post-weighed in a temperature and relative-humidity controlled glove box (PlasLabs Model 890 THC, Lansing, MI, USA) to determine total PM_2.5_ mass. Reflectometry for BC absorbance was performed using an EEL43M Smokestain Reflectometer (Diffusion Systems, Ltd., London, UK). Inductively-coupled plasma mass spectrometry (ICP-MS) analyses were conducted for elemental constituents by the Wisconsin State Laboratory of Hygiene following documented protocols (ESS INO Method 400.4; EPA Method 1638) [25]. For OC and EC, thermal-optical reflectance was run by Desert Research Institute (DRI, Reno, NV, USA) [26], and TD-GC-MS was conducted for organic compounds [27,28].

### 2.7. Quality Assurance and Quality Control

Multiple laboratory and field blanks were collected each session to quantify contamination, on both filter types. Pump flow rates were calibrated to 4.0 LPM, temperature-adjusted based on average forecasted temperatures for the sampling period, and re-assessed after sampling completion. All sampling pumps met acceptable pre- and post-collection flow rates, within ± 5% of 4.0 LPM. A small number of sites (*n* = 2 for Teflon filters, *n* = 2 for quartz filters) were re-sampled due to equipment failure during the scheduled session. Field blanks from the same session were used to blank-correct all pollutant concentrations. Data completeness was 96 to 100% for interval- and season-specific sampling campaigns, with no statistical outliers (outside of ±3 standard deviations). One summer work-week PM_2.5_ sample was lost due to field equipment error. One additional trace element sample was lost due to analytic equipment error.

### 2.8. GIS-Based Source Density Indicators

We examined both rush-hour and work-week data using the GIS-based source covariates and modifiers reported in Tunno et al. [1]. All covariates were created using ArcInfo Version 10.2 (ESRI, Redlands, CA, USA). Buffer-based covariates were created for concentric radial buffers surrounding each monitoring location (25 to 200 m), to assess impacts of sources at various distances on observed pollutant concentrations. Buffers larger than 200 m were not considered, because these buffers overlapped between neighboring sites, dampening variance across sites.

### 2.9. Temporal Adjustment

Using both the rural and urban reference sites, we temporally-adjusted all samples to account for between-session variability driven by time-varying meteorology or long-range pollutant transport. We estimated the expected seasonal average concentration at each site by dividing the observed concentration by the session-specific average concentration from both reference sites, then multiplied the result by the seasonal average concentration from both reference sites, as detailed in Shmool et al. [29].

### 2.10. Statistical Analysis

We calculated descriptive statistics for PM_2.5_, BC, total EC and OC, trace elements previously associated with diesel emissions (Al, Ca, Fe), and organic compounds, and compared temporally-adjusted pollutant concentration distributions across the source indicator strata used for site selection. We compared pollutant concentrations within sites, by season and by sampling interval (i.e., rush-hour vs work-week), using paired *t*-tests. We identified potential statistical outliers (outside of mean ± 3 standard deviations) within each sampling session. Before LUR modeling, bivariate source-pollutant correlations were performed to select candidate source terms, and each source indicator (Table 1) was assessed individually as a predictor of each temporally-adjusted pollutant concentration. Data analysis and model-building were performed separately for each pollutant by rush and work-week programs, and for summer and winter seasons.

LUR models were derived, for each of the pollutants described above, using manual forward step-wise linear regression, predicting raw pollutant concentrations for the summer and winter seasons, using an adapted version of the modeling approach in Tunno et al. [9]. In model building, temporal trends in pollutant concentrations were incorporated first into LUR models, using the session-specific mean reference concentration. Source terms with the strongest univariate correlation to the temporally-adjusted pollutant were then incorporated, individually in descending order by strength of the bivariate correlation. Regression models were sequentially fit to assess overall model improvement at each stage, using the coefficient of determination (R^2^), and removing non-significant covariates in order of descending *p*-value, until all independent terms were significant (*p* < 0.05). Covariates were removed, at any stage, if the variance inflation factor (VIF) became greater than 2.0, to reduce multicollinearity between variables.

LUR model residuals were mapped to identify systematic spatial variation and locations poorly predicted by LUR, suggesting the need to identify and incorporate additional covariates. Semivariograms of residuals were created in GIS, and spatial autocorrelation in residuals was tested using the Moran’s I statistic.

### 2.11. Sensitivity Analyses

Covariate selection was sensitivity-tested using scatterplots to assess fit between each significant predictor and raw pollutant concentrations, to ensure that selected candidate covariates captured variability across the range of concentrations, and that associations were not reliant on outliers or influential points. Tree structures and Random Forest automated methods were performed to corroborate covariate selection. A scatterplot of each retained term was tested against the residual of the prior model in the sequential model-building process to check for outliers and overall fit. We examined model residuals to ensure normality. Backwards elimination, from multivariate linear models including all source covariates with significant bivariate associations with temporally-adjusted concentrations, was used to further corroborate model structure and covariate selection. For validation of LUR predictions, a random 20% of sites (*n* = 5) were removed from the analysis, and the LUR model was re-fit and used to predict pollutant concentrations at withheld sites. Analyses were performed in SAS v 9.4 (SAS Institute Inc., Cary, NC, USA), ArcInfo, v 10.1 (ESRI, Redlands, CA, USA), R statistical software v 3.1.2, and Excel 2010 (Microsoft, Inc. Redmond, WA, USA).

## 3. Results

### 3.1. Summary Statistics and Spatial Patterns

**Figure 2:**
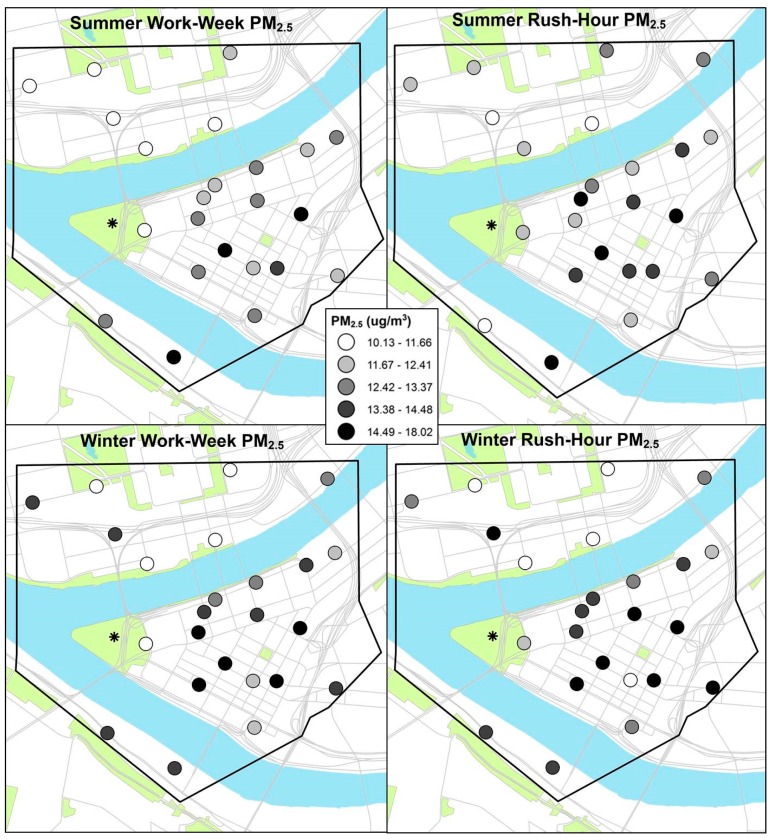
Paired work-week and rush-hour temporally-adjusted PM_2.5_ concentrations (in quintiles) across 24 distributed monitoring sites for summer (**top**) and winter (**bottom**) sampling.

**Figure 3:**
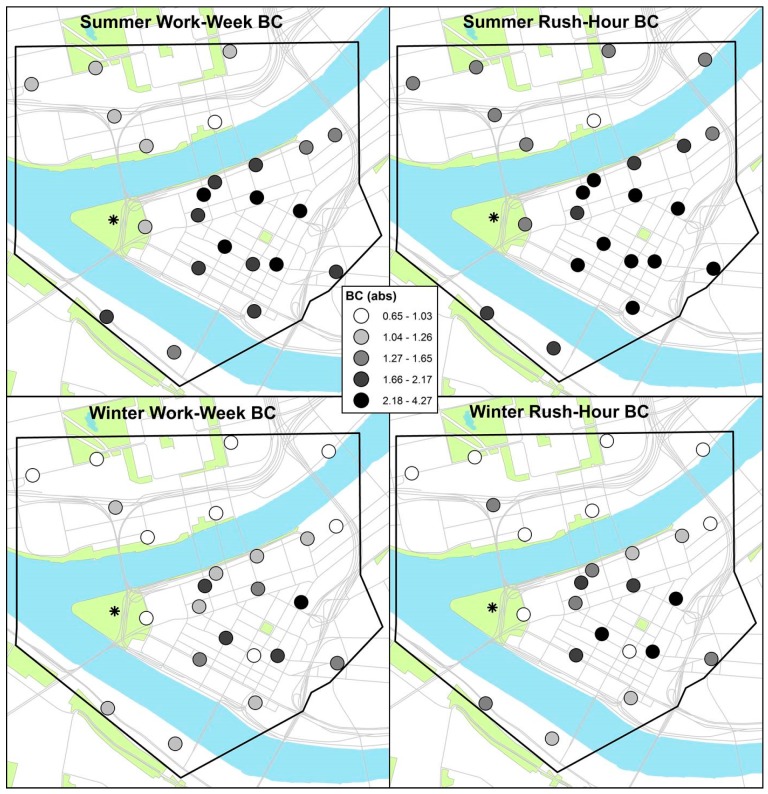
Paired work-week and rush-hour BC absorbance (in quintiles) across 24 distributed monitoring sites for summer (**top**) and winter (**bottom**) sampling.

We found no significant differences in total PM_2.5_ concentrations, either by season or sampling scheme. Spatial patterning in PM_2.5_ for each season was similar, with generally higher concentrations in the center of downtown. Notably, differences in concentrations of PM_2.5_ were greater across sites than between seasons. In both seasons, we found higher average PM_2.5_ concentrations during rush-hours than over the full work-week (Table 1). There were no apparent spatial differences in PM_2.5_ or constituent concentrations by sampling scheme or season (Figure 2 and Figure 3, other constituent maps not shown).

### 3.2. Comparisons between Sampling Schemes

During winter, we found greater concentrations of *diesel-related* (i.e., BC, EC, benz(a)anthracene, benzo(e)pyrene, and chrysene) and *traffic/road dust-related constituents* (e.g., copper, manganese, potassium, zinc) in rush-hour vs. full work-week samples (Table 1). Conversely, hopane (*diesel*) and arsenic (*coal*) concentrations were higher in work-week than rush-hour samples.

During summer, we found greater concentrations of some diesel-related constituents (i.e., BC, fluoranthene, pyrene, cholestane) in rush-hour vs. work-week samples, though some (i.e., hopanes) were higher in work-week samples (Table 2).

### 3.3. Comparisons between Seasons

Concentrations of BC, EC, and OC were higher in summer than winter, in both rush-hour and work-week samples (Supplementary Materials Tables S1 and S2).

In rush-hour samples, concentrations of *coal-related* elements (i.e., As, Se) were higher during summer. *Motor-vehicle-related elements* (i.e., calcium, phosphorus, potassium, zinc) and *diesel-related* organics (i.e., benzo(e)pyrene, benzo(ghi)perylene, pyrene, total PAHs) were higher during winter (Supplementary Materials Table S1).

Likewise, in the work-week samples, concentrations of *motor vehicle-related* zinc, and *diesel-related* organics (i.e., benzo(e)pyrene, benzo(ghi)perylene, chrysene, fluoranthene, pyrene, total PAHs) were higher during winter than summer (Supplementary Materials Table S2).

### 3.4. LUR Models

For PM_2.5_ and EC, the only significant source covariate retained in LUR models, for both seasons and sampling schemes, was mean bus density within 200 m of the sampling site (Table 3 and Table 4). For BC, mean bus density was the strongest term for both seasons and sampling schemes; truck density within 200 m explained additional spatial variability in summer.

For OC, bus stop use (number of stops per day) was the only significant predictor, for both sampling schemes, during winter (Table 3). During summer, truck density explained variability in rush-hour samples; commercial land use and buildings aspect ratio explained variability in work-week samples (Table 4).

For total PAHs, bus-related covariates were significant for both seasons and schemes (Table 3 and Table 4). For total hopanes, bus density explained spatial variance in full work-week samples, in both seasons. For rush-hour samples, no source covariates predicted spatial variance during winter; only truck density explained spatial variance in summer.

For the elemental tracers (Al, Ca, Fe), during winter, no covariates explained spatial variance in Al or Fe, using either sampling scheme (Table 3). For Ca, distance to primary road explained variability in rush-hour samples; bus density explained variation in work-week samples. During summer, for all three elements, truck density explained spatial variation in rush-hour samples (Table 4). No source covariates explained spatial variation in summer work-week samples of Al or Ca; length of roadway explained spatial variability in work-week Fe.

### 3.5. Sensitivity Analyses

Scatterplots revealed that final models were not driven by outliers or influential points. Tree structures and Random Forest automated methods corroborated the covariates retained in final models. Moran’s *I* results indicated no spatial autocorrelation in model residuals across distributed monitoring sites. Removal of a random subset (20%) of monitoring sites did not significantly change any of the models, and predicted concentrations at the withheld sites were within 10% of the measured concentrations.

## 4. Discussion

Air pollution saturation studies are now commonly used in exposure assessment and environmental epidemiology, but few have been able to capture spatial variation during selected hours of the day (e.g., rush-hours, inversion events [9]), largely due to technical and equipment limitations. Further, relatively few studies to date have been able to incorporate organic pollutants into spatial saturation studies. We sampled gasoline and diesel-related organic and elemental constituents during rush-hour and work-week hours, to disentangle a complex mix of sources impacting spatial variation in air pollution exposures across an urban core.

This study is the first, to our knowledge, to explicitly capture and compare spatial variation in multiple pollutants during high diesel source-intensity hours (“rush hours”) vs. the overall work-week. We hypothesized heightened concentrations and sharper spatial contrasts in multiple pollutants during the rush hours, and expected to identify source terms which differently explained spatial variation in multiple pollutant concentrations during these different hours of the day. Rush-hour sampling indicated non-significantly higher PM_2.5_ concentrations compared to work-week sampling, in both seasons, with *no evident difference* in spatial patterns—possibly suggested that rush-hours emissions accounted for a large portion of total PM_2.5_ concentrations and spatial variance across the downtown core.

We observed higher concentrations of traffic-related elemental and organic constituents during rush-hours, relative to the work-week, again with similar spatial patterning using both sampling schemes. The differences between rush hours and the full work week were stronger for BC and other components than for PM_2.5_—possibly because BC and other components may be more specific to traffic and diesel emissions than is total PM_2.5_ [30,31].

GIS-based source terms explained similar variability (R^2^) during the rush hours and full work-week, possibly suggesting that the bulk of spatial contrast in diesel-related pollutants may be explained during rush hours. Bus traffic explained the bulk of spatial variance in rush-hour concentrations, and accounted for some spatial variation in work-week samples. Higher concentrations of coal-related elements during work-week, relative to the rush hours, suggests that non-traffic industrial sources may have a greater relative influence on pollution exposure patterns during non-rush-hours.

Comparing between seasons, PM_2.5_ concentrations did not differ significantly, possibly pointing to the consistency of commuting traffic, working hours, and bus and truck traffic year-round. For BC, EC, and OC, we found higher concentrations during summer compared to winter. The differences between rush hours and the full work week were stronger for BC than for PM_2.5_—possibly because BC may be more specific to traffic and diesel emissions than is total PM_2.5_.

Though we hypothesized greater spatial contrasts and different source contributions during rush hours, LUR models for both schemes were comparable for PM_2.5_ and BC, with bus density as the lone significant predictor. The significance of the bus indicators in predicting concentrations of many pollutants may indicate a greater accuracy in this GIS variable, compared to other source terms, or a particularly strong impact of bus-related emissions. This same pattern held true for total EC rush-hour models in both seasons, as well as winter total OC models, suggesting slightly higher pollutant concentrations and spatial contrasts during rush hours.

We may have observed lesser spatial contrast, and weaker fits for GIS-based source terms, during winter than summer due to a lower mixing height, cooler ambient temperature, and/or greater frequency of inversion events (possibly flattening out spatial contrasts in pollution across this small area of relatively uniform elevation).

There are several limitations to our study, including the lack of downtown-area meteorological data (i.e., windspeed and direction), limiting our ability to account for complex dispersal patterns in and around urban street canyons. In addition, the majority of GIS-based source terms available were based on annual-average source intensity (e.g., annual-average daily traffic)—limiting our ability to identify short-term changes in local emissions during our sampling period. Finally, limited equipment availability required us to sample different sites over several weeks, and to temporally-adjust all concentrations prior to LUR modelling and comparison of spatial patterns. As higher diesel-related exposures are generally found in densely-populated urban areas, diesel particulate matter (DPM) can have a tremendous impact on public health. By performing a saturation sampling campaign across the downtown area during specific time periods (i.e., rush hours), we were able to identify locations and times of peak pollution exposures, and were able to identify potential contributing sources using land use regression modelling. New policies could also be implemented to target specific sources found in the LUR models (e.g., bus routes), to decrease pollutant exposures.

Future studies should consider sampling, as possible, during high- and low-source intensity hours, and include tracers for a wider variety of sources; our study could not, for example, examine impacts of railroads, barges, and other sources, although they may impact upon total diesel-related exposures, and contribute differently to the concentrations of these various constituents. Furthermore, the Pittsburgh Port Authority is changing the majority of their bus fleet to natural gas in the coming years; validation analyses for the effectiveness of this intervention in reducing local concentrations could include re-sampling using this or a similar spatial saturation design.

## 5. Conclusions

This study is among the most densely-saturated monitoring campaigns, to our knowledge, and was specifically designed to capture spatial variation in pollution during selected hours of the day, incorporating both elemental and organic tracers of diesel-related emissions into our analysis. This method could be replicated across other urban areas to assess whether spatial variation differs by time of day. Future studies could focus on variability in constituents, with an emphasis on local meteorological phenomenon and its impact on spatial gradients in exposure.

Mean bus density was the strongest LUR predictor, regardless of sampling interval, underscoring the significant contribution of bus exhaust to overall pollutant concentrations. While rush-hour sampling produced slightly higher PM_2.5_ concentrations, there were no evident differences in spatial patterns, compared to work-week sampling, and rush-hour and work-week LUR models explained similar proportions of variability in concentrations in each season. We found substantial spatial variability in most trace elements and organic compounds, with comparable spatial patterns, using both sampling paradigms. Overall, we found higher concentrations of traffic-related trace elements and organic compounds in the rush-hour samples, and higher concentrations of coal-related elements (e.g., As, Se) in the work-week samples. Together, these results indicate that vehicular source patterning during rush hours may account for a substantial portion of overall spatial variation in exposures.

## Figures and Tables

**Table 1:** Temporally-adjusted winter pollutant concentrations across 24 distributed sites, by sampling interval. Concentrations which differed significantly between sampling schemes are shown in bold (*p* < 0.05).

Pollutant	Rush-Hour Sampling		Work-Week Sampling		*p*-Value between Programs
	Mean (SD)	Median	Mean (SD)	Median	
PM_2.5_ (µg/m^3^)	13.5 (1.63)	13.4	13.2 (1.67)	13.6	0.07
**BC (abs)**	**1.39 (0.58)**	**1.22**	**1.25 (0.47)**	**1.10**	**0.0001**
**Total EC (µg/m^3^)**	**1.57 (0.74)**	**1.48**	**1.43 (0.62)**	**1.28**	**0.01**
Total OC (µg/m^3^)	2.29 (0.62)	2.03	2.45 (0.77)	2.37	0.11
**Diesel Tracers (ng/m^3^):**					
Al	28.3 (14.4)	23.8	27.8 (17.1)	24.0	0.90
Ca	104.1 (77.85)	86.08	86.3 (48.3)	92.0	0.31
Fe	115.8 (49.47)	104.9	109.2 (72.9)	97.4	0.62
**Other Tracer Elements (ng/m^3^):**					
**As**	**0.13 (0.10)**	**0.10**	**0.30 (0.28)**	**0.24**	**0.01**
Ba	4.72 (2.16)	3.98	4.30 (2.91)	3.73	0.37
Cr	1.26 (0.42)	1.22	1.09 (0.43)	1.15	0.10
**Cu**	**5.05 (2.04)**	**4.36**	**4.30 (2.24)**	**4.15**	**0.04**
**K**	**48.9 (12.19)**	**49.6**	**42.7 (16.3)**	**41.5**	**0.04**
Mg	31.9 (69.20)	14.9	16.9 (11.5)	16.2	0.32
**Mn**	**5.65 (2.30)**	**5.37**	**4.55 (2.10)**	**4.44**	**0.004**
Mo	21.7 (97.61)	1.77	32.3 (106.9)	0.89	0.49
Ni	2.14 (4.21)	0.37	0.69 (0.79)	0.40	0.11
P	4.98 (1.31)	4.92	4.43 (1.47)	4.31	0.08
Pb	2.54 (0.52)	2.54	2.44 (0.90)	2.53	0.56
S	587.4 (110.60)	593.9	568.4 (178.0)	602.8	0.61
Sb	0.94 (0.30)	0.85	0.82 (0.33)	0.83	0.07
**Se**	**0.41 (1.86)**	**0.75**	**1.62 (1.50)**	**1.28**	**0.03**
Sr	0.56 (0.24)	0.51	0.54 (0.28)	0.54	0.66
V	0.22 (0.04)	0.23	0.23 (0.07)	0.23	0.73
**Zn**	**26.9 (9.87)**	**27.1**	**19.1 (9.17)**	**17.8**	**0.001**
**PAHs (ng/m^3^):**					
**Benz(a)anthracene**	**0.08 (0.04)**	**0.07**	**0.07 (0.03)**	**0.06**	**0.003**
Benzo(a)pyrene	0.03 (0.04)	0.01	0.04 (0.03)	0.03	0.77
**Benzo(e)pyrene**	**0.08 (0.03)**	**0.08**	**0.04 (0.01)**	**0.04**	**0.01**
Benzo(ghi)fluoranthene	0.04 (0.02)	0.04	0.06 (0.02)	0.06	0.14
Benzo(ghi)perylene	0.03 (0.01)	0.03	0.04 (0.03)	0.03	0.11
**Chrysene**	**0.23 (0.07)**	**0.19**	**0.19 (0.08)**	**0.16**	**<0.0001**
Fluoranthene	0.20 (0.07)	0.19	0.20 (0.08)	0.17	0.53
Indeno(1,2,3-cd)pyrene	0.01 (0.002)	0.01	0.01 (0.02)	0	0.98
Pyrene	0.16 (0.05)	0.15	0.17 (0.07)	0.15	0.43
Total PAHs	0.71 (0.21)	0.66	0.72 (0.18)	0.64	0.74
**Hopanes (ng/m^3^):**					
**Total hopanes**	**0.15 (0.11)**	**0.12**	**0.19 (0.11)**	**0.16**	**0.05**

Total steranes samples were below LOD, using both sampling schemes.

**Table 2:** Temporally-adjusted summer pollutant concentrations across 24 distributed sites, by sampling interval. Concentrations which differed significantly between sampling schemes are shown in bold (*p* < 0.05).

Pollutant	Rush-Hour Sampling		Work-Week Sampling		*p*-Value between Programs
	Mean (SD)	Median	Mean (SD)	Median	
PM_2.5_ (µg/m^3^) ^a^	13.1 (1.69)	12.4	12.8 (1.98)	12.6	0.18
**BC (abs) ^a^**	**2.09 (0.76)**	**1.76**	**1.83 (0.64)**	**1.97**	**<0.0001**
Total EC (µg/m^3^)	1.98 (0.89)	1.57	1.85 (0.76)	1.85	0.13
Total OC (µg/m^3^)	2.87 (0.91)	2.51	2.65 (0.56)	2.64	0.16
**Diesel Tracers ^b^:**					
Al	35.2 (35.89)	23.8	52.2 (44.6)	36.16	0.18
Ca	63.6 (58.10)	52.4	83.7 (86.3)	51.20	0.22
Fe	106.2 (48.82)	107.8	121.9 (85.3)	106.4	0.53
**Tracers of Other Sources ^b^:**					
As	0.60 (0.18)	0.60	0.59 (0.21)	0.62	0.84
Ba	4.82 (2.93)	4.38	6.55 (6.94)	3.91	0.37
Cr	1.30 (0.69)	1.29	1.51 (1.25)	1.33	0.53
Cu	5.09 (2.88)	4.50	5.59 (4.03)	4.60	0.69
K	34.8 (18.87)	32.6	38.5 (21.4)	37.4	0.56
Mg	10.2 (6.59)	9.74	17.7 (19.8)	8.23	0.12
Mn	4.50 (2.50)	4.31	4.91 (2.96)	4.47	0.60
Mo	1.92 (0.79)	1.88	2.14 (1.32)	1.95	0.42
Ni	0.58 (0.36)	0.52	0.67 (0.52)	0.58	0.49
P	3.63 (1.52)	3.59	3.99 (2.25)	3.65	0.52
Pb	2.39 (1.01)	2.40	2.61 (1.64)	2.37	0.70
S	571.2 (216.59)	615.9	781.1 (424.3)	771.9	0.06
Sb	1.14 (0.60)	0.98	1.24 (0.87)	1.03	0.63
Se	1.21 (0.37)	1.28	1.24 (0.33)	1.32	0.82
Sr	0.59 (0.31)	0.57	0.61 (0.41)	0.54	0.94
V	0.30 (0.05)	0.30	0.30 (0.04)	0.30	0.61
Zn	11.1 (5.14)	10.7	13.2 (8.4)	11.7	0.38
**PAHs (ng/m^3^):**					
Benzo(a)pyrene	0.07 (0.06)	0.05	0.04 (0.01)	0.04	0.15
Benzo(e)pyrene	0.03 (0.02)	0.03	0.03 (0.01)	0.03	0.22
Benzo(ghi)perylene	0.01 (0.01)	0.01	0.01 (0.01)	0.01	0.58
Chrysene	N/A	N/A	0.04 (0.02)	0.03	N/A
**Fluoranthene**	**0.17 (0.15)**	**0.12**	**0.11 (0.09)**	**0.08**	**0.02**
Indeno(1,2,3-cd)pyrene	0.01 (0.01)	0.01	0.01 (0.01)	0.01	0.52
**Pyrene**	**0.08 (0.08)**	**0.04**	**0.05 (0.05)**	**0.03**	**0.03**
Total PAHs	0.22 (0.22)	0.16	0.14 (0.15)	0.09	0.07
**Hopanes (ng/m^3^):**					
**Total hopanes**	**0.13 (0.10)**	**0.11**	**0.18 (0.14)**	**0.13**	**0.03**
**Steranes (ng/m^3^):**					
**Cholestane**	**0.03 (0.02)**	**0.03**	**0.02 (0.02)**	**0.02**	**0.02**

Benz(a)anthracene and benzo(ghi)fluoranthene samples were below LOD for both programs; ^a^ One PM/BC/elemental measurement was lost due to chrontroller error during work-week program; ^b^ For the work-week program, one elemental sample was lost due to sampling instrument failure; one elemental sample lost due to analytic instrument failure.

**Table 3:** Winter rush-hour and work-week LUR summary for all modeled pollutants. All models include a temporal term (mean reference site concentration for the session).

Pollutant	Rush-Hour Samples				Work-Week Samples			
	Spatial Covariate(s)	β (*p*-Value)	Conc. Incr. per IQR	R^2^	Spatial Covariate(s)	β (*p*-Value)	Conc. Incr. per IQR	R^2^
PM_2.5_ (µg/m^3^)	Bus Density (200 m)	9.0 × 10^−9^ (0.02)	1.05	0.78	Bus Density (200 m)	1.1 × 10^−8^ (0.01)	1.29	0.77
BC (abs)	Bus Density (200 m)	5.0 × 10^−9^ (<0.0001)	0.58	0.61	Bus Density (200 m)	4.0 × 10^−9^ (<0.0001)	0.47	0.63
EC (µg/m^3^)	Bus Density (50 m)	4.0 × 10^−9^ (<0.0001)	0.59	0.72	Bus Density (50 m)	4.0 × 10^−9^ (<0.0001)	0.59	0.76
OC (µg/m^3^)	Bus stop events/day (175 m)	1.7 × 10^−4^ (<0.0001)	0.58	0.74	Bus stop events/day (175 m)	2.1 × 10^−4^ (<0.0001)	0.72	0.69
Total PAHs (ng/m^3^)	Bus Density (50 m)	1.0 × 10^−9^ (<0.0001)	0.15	0.88	Bus Density (50 m)	1.0 × 10^−9^ (< 0.0001)	0.15	0.89
Hopanes (ng/m^3^)	-	-	-	-	Bus Density (50 m)	1.0 × 10^−9^ (0.02)	0.15	0.55
Al (ng/m^3^)	-	-	-	0.30	-	-	-	0.18
Ca (ng/m^3^)	Distance to primary road	−0.29 (0.02)	8.91	0.41	Bus Density (50 m)	2.0 × 10^−7^ (0.01)	29.7	0.38
Fe (ng/m^3^)	-	-	-	0.35	-	-	-	0.07

**Table 4:** Summer rush-hour and work-week LUR summary for all modeled pollutants. All models include a temporal term (mean reference site concentration for the session).

Pollutant	Rush-Hour Samples				Work-Week Samples			
	Spatial Covariate(s)	β (*p*-Value)	Conc. Incr. per IQR	R^2^	Spatial Covariate(s)	β (*p*-Value)	Conc. Incr. per IQR	R^2^
PM_2.5_ (µg/m^3^)	Bus Density (200 m)	1.3 × 10^−8^ (0.001)	2.16	0.72	Bus Density (50 m)	7.0 × 10^−9^ (<0.0001)	1.16	0.69
BC (abs)	Bus Density (200 m)	4.0 × 10^−9^ (0.02)	0.66	-	Bus Density (200 m)	3.4 × 10^−9^ (0.02)	0.50	-
	Truck Density (200 m)	6.8 × 10^−5^ (0.005)	0.40	0.75	Truck Density (200 m)	5.7 × 10^−5^ (0.01)	0.34	0.71
EC (µg/m^3^)	Bus Density (200 m)	7.0 × 10^−9^ (<0.0001)	1.16	0.62	Bus Density (200 m)	7.0 × 10^−9^ (<0.0001)	1.16	0.62
OC (µg/m^3^)	Truck Density (150 m)	3.2 × 10^−5^ (0.03)	0.24	0.49	Commercial land use (150 m)	2.0 × 10^−6^ (0.001)	0.51	
	-	-	-	-	Buildings aspect ratio	0.064 (0.06)	0.27	0.63
Total PAHs (ng/m^3^)	Bus stop events/day (200 m)	8.0 × 10^−5^ (0.002)	0.37	0.61	Bus Density (200 m)	2.0 × 10^−9^ (0.02)	0.23	0.66
Hopanes (ng/m^3^)	Truck Density (200 m)	2.2 × 10^−5^ (0.002)	0.13	0.50	Bus Density (200 m)	1.8 × 10^−9^ (<0.0001)	0.21	0.53
Al (ng/m^3^)	Truck Density (200 m)	1.8 × 10^−3^ (0.02)	10.6	0.39	-	-	-	0.02
Ca (ng/m^3^)	Truck Density (200 m)	2.5 × 10^−3^ (0.02)	14.7	0.33	-	-	-	0.07
Fe (ng/m^3^)	Truck Density (200 m)	4.0 × 10^−3^ (0.02)	23.5	0.37	Primary & secondary road length (25 m)	0.34 (0.01)	7.82	0.40

## Supplementary Materials

The following are available online at http://www.mdpi.com/1660-4601/15/9/1968/s1, Table S1: Summary and comparison of rush-hour temporally-adjusted pollutant concentrations by winter and summer seasons, Table S2: Summary and comparison of work-week temporally-adjusted pollutant concentrations by winter and summer seasons.
