# Supplementary material for: Spatial Patterns in Rush-Hour vs. Work-Week Diesel-Related Pollution across a Downtown Core
Source: Int J Environ Res Public Health. 2018 Sep 10;15(9):1968. doi: 10.3390/ijerph15091968 (PMC6164514; doi:10.3390/ijerph15091968)
Supplement: Supplementary file 1 [file ijerph-15-01968-s001.pdf]

Supporting Information:

Table S1. Summary and comparison of rush-hour temporally-adjusted pollutant concentrations by winter and summer seasons.

	**Summer Season**		**Winter Season**		
**Pollutant**	**Mean (SD)**	**Median**	**Mean (SD)**	**Median**	**p-value between programs**
$PM_{2.5}$ (μg/m³)	13.12 (1.69)	12.61	13.45 (1.63)	13.41	0.29
BC (abs)	2.09 (0.76)	1.97	1.39 (0.58)	1.22	**<0.0001**
Total EC (μg/m³)	1.98 (0.89)	1.85	1.57 (0.74)	1.48	**0.03**
Total OC (μg/m³)	2.87 (0.91)	2.64	2.29 (0.62)	2.03	**0.003**
**Diesel Tracers (ng/m³):**					
Al	35.24 (35.89)	23.83	28.25 (14.43)	23.84	0.41
Ba	4.82 (2.93)	4.38	4.72 (2.16)	3.98	0.89
**Ca**	**63.63 (58.10)**	**52.41**	**104.10 (77.85)**	**86.08**	**0.06**
Cr	1.30 (0.69)	1.29	1.26 (0.42)	1.22	0.81
Cu	5.09 (2.88)	4.50	5.05 (2.04)	4.36	0.95
Fe	106.17 (48.82)	107.79	115.81 (49.47)	104.85	0.54
Mg	10.19 (6.59)	9.74	31.86 (69.20)	14.86	0.15
**P**	**3.63 (1.52)**	**3.59**	**4.98 (1.31)**	**4.92**	**0.004**
S	571.15 (216.59)	615.94	587.35 (110.60)	593.93	0.75
**Zn**	**11.07 (5.14)**	**10.70**	**26.86 (9.87)**	**27.06**	**<0.0001**
**Tracers of Other Sources (ng/m³):**					
**As**	**0.60 (0.18)**	**0.60**	**0.13 (0.10)**	**0.10**	**<0.0001**
**K**	**34.78 (18.87)**	**32.57**	**48.86 (12.19)**	**49.55**	**0.002**
Mn	4.50 (2.50)	4.31	5.65 (2.30)	5.37	0.13
Mo	1.92 (0.79)	1.88	21.67 (97.61)	1.77	0.33
Ni	0.58 (0.36)	0.52	2.14 (4.21)	0.37	0.09
Pb	2.39 (1.01)	2.40	2.54 (0.52)	2.54	0.55
Sb	1.14 (0.60)	0.98	0.94 (0.30)	0.85	0.17
**Se**	**1.21 (0.37)**	**1.28**	**0.41 (1.86)**	**0.75**	**0.06**
Sr	0.59 (0.31)	0.57	0.56 (0.24)	0.51	0.82
**V**	**0.30 (0.05)**	**0.30**	**0.22 (0.04)**	**0.23**	**<0.0001**
**PAHs (ng/m³):**					
Benz[a]anthracene	N/A	N/A	0.08 (0.04)	0.07	N/A
Benzo[a]pyrene	0.07 (0.06)	0.05	0.03 (0.04)	0.01	0.13
**Benzo[e]pyrene**	**0.03 (0.02)**	**0.03**	**0.08 (0.03)**	**0.08**	**0.01**
Benzo[ghi]fluoranthene	N/A	N/A	0.04 (0.02)	0.04	N/A
**Benzo[ghi]perylene**	**0.01 (0.01)**	**0.01**	**0.03 (0.01)**	**0.03**	**0.04**
Chrysene	N/A	N/A	0.23 (0.07)	0.19	N/A

Fluoranthene	0.17 (0.15)	0.12	0.20 (0.07)	0.19	0.27
Indeno[1,2,3-cd]pyrene	0.01 (0.01)	0.01	0.01 (0.002)	0.01	N/A
**Pyrene**	**0.08 (0.08)**	**0.04**	**0.16 (0.05)**	**0.15**	**0.001**
**Total PAHs**	**0.22 (0.22)**	**0.16**	**0.71 (0.21)**	**0.66**	**<0.0001**
**Hopanes (ng/m^3):**					
Total hopanes	0.13 (0.10)	0.11	0.15 (0.11)	0.12	0.57
**Steranes (ng/m^3):**					
**Cholestane**	**N/A**	**N/A**	**N/A**	**N/A**	**<0.0001**

Table S2. Summary and comparison of work-week temporally-adjusted pollutant concentrations by winter and summer seasons.

	**Summer Season**		**Winter Season**		
**Pollutant**	**Mean (SD)**	**Median**	**Mean (SD)**	**Median**	**p-value between programs**
$PM_{2.5}$ (μg/m^3)[a]	12.84 (1.98)	12.41	13.24 (1.67)	13.63	0.24
BC (abs)[a]	1.83 (0.64)	1.76	1.25 (0.47)	1.10	**<0.0001**
Total EC (μg/m^3)	1.85 (0.76)	1.57	1.43 (0.62)	1.28	**0.001**
Total OC (μg/m^3)	2.65 (0.56)	2.51	2.45 (0.77)	2.37	0.10
**Diesel Tracers (ng/m^3)[b]:**					
**Al**	**52.23 (44.58)**	**36.16**	**27.83 (17.06)**	**24.04**	**0.02**
Ba	6.55 (6.94)	3.91	4.30 (2.91)	3.73	0.13
Ca	83.72 (86.34)	51.20	86.28 (48.32)	92.02	0.89
Cr	1.51 (1.25)	1.33	1.09 (0.43)	1.15	0.09
**Cu**	**5.59 (4.03)**	**4.60**	**4.30 (2.24)**	**4.15**	**0.06**
Fe	121.94 (85.25)	106.40	109.21 (72.89)	97.38	0.59
Mg	17.69 (19.77)	8.23	16.89 (11.54)	16.19	0.80
P	3.99 (2.25)	3.65	4.43 (1.47)	4.31	0.42
**S**	**781.12 (424.32)**	**771.86**	**568.44 (178.00)**	**602.76**	**0.04**
**Zn**	**13.15 (8.38)**	**11.67**	**19.05 (9.17)**	**17.78**	**0.03**
**Tracers of Other Sources (ng/m^3)[b]:**					
**As**	**0.59 (0.21)**	**0.62**	**0.30 (0.28)**	**0.24**	**0.003**
K	38.46 (21.37)	37.40	42.66 (16.31)	41.50	0.40
Mn	4.91 (2.96)	4.47	4.55(2.10)	4.44	0.44
Mo	2.14 (1.32)	1.95	32.26 (106.88)	0.89	0.18
Ni	0.67 (0.52)	0.58	0.69 (0.79)	0.40	0.73
Pb	2.61 (1.64)	2.37	2.44 (0.90)	2.53	0.64
**Sb**	**1.24 (0.87)**	**1.03**	**0.82 (0.33)**	**0.83**	**0.03**
Se	1.24 (0.33)	1.32	1.62 (1.50)	1.28	0.18
Sr	0.61 (0.41)	0.54	0.54 (0.28)	0.54	0.43
**V**	**0.30 (0.04)**	**0.30**	**0.23 (0.07)**	**0.23**	**0.0004**
**PAHs (ng/m^3):**					

Benz[a]anthracene	N/A	N/A	0.07 (0.03)	0.06	N/A
Benzo[a]pyrene	0.04 (0.01)	0.04	0.04 (0.03)	0.03	0.14
**Benzo[e]pyrene**	**0.03 (0.01)**	**0.03**	**0.04 (0.01)**	**0.04**	**0.02**
Benzo[ghi]fluoranthene	N/A	N/A	0.06 (0.02)	0.06	N/A
**Benzo[ghi]perylene**	**0.01 (0.01)**	**0.01**	**0.04 (0.03)**	**0.03**	**0.05**
**Chrysene**	**0.04 (0.02)**	**0.03**	**0.19 (0.08)**	**0.16**	**0.0002**
**Fluoranthene**	**0.11 (0.09)**	**0.08**	**0.20 (0.08)**	**0.17**	**0.002**
Indeno[1,2,3-cd]pyrene	0.01 (0.01)	0.01	0.01 (0.02)	0	0.92
**Pyrene**	**0.05 (0.05)**	**0.03**	**0.17 (0.07)**	**0.15**	**0.0003**
**Total PAHs**	**0.14 (0.15)**	**0.09**	**0.72 (0.18)**	**0.64**	**<0.0001**
**Hopanes (ng/m^3):**					
Total hopanes	0.18 (0.14)	0.13	0.19 (0.11)	0.16	0.77
**Steranes (ng/m^3):**					
**Cholestane**	**0.02 (0.02)**	**0.02**	**N/A**	**N/A**	**<0.0001**

[a]One measurement lost due to chrontroller failure in summer.

[b]One sample lost due to chrontroller error; one sample lost due to microwave bomb failure in summer.
